# Detection of microsatellite instability high (MSI-H) status by targeted plasma-based genotyping in metastatic breast cancer

**DOI:** 10.1038/s41523-022-00490-2

**Published:** 2022-11-04

**Authors:** Neelima Vidula, Andrew Lipman, Shumei Kato, Caroline Weipert, Katherine Hesler, Georges Azzi, Ahmed Elkhanany, Dejan Juric, Estelamari Rodriguez, Colleen Faulkner, Paul Makhlouf, Kristin Price, Joyce O’Shaughnessy, Aditya Bardia

**Affiliations:** 1 Massachusetts General Hospital Cancer Center, Harvard Medical School, Boston, MA USA; 2 Florida Cancer Specialists, Sun City Center, FL USA; 3 University of California San Diego, San Diego, CA USA; 4 Guardant Health, Redwood, CA USA; 5 Bienes Cancer Center, Fort Lauderdale, FL USA; 6 University of Alabama at Birmingham, Birmingham, AL USA; 7 Sylvester Comprehensive Cancer Center, University of Miami Miller School of Medicine, Miami, FL USA; 8 Texas Oncology-Baylor Charles A. Sammons Cancer Center, Dallas, TX USA

**Keywords:** Breast cancer, Breast cancer

## Abstract

We evaluate microsatellite instability-high (MSI-H) status with cell-free DNA (cfDNA) in metastatic breast cancer (MBC) and the association with clinico-genomic characteristics. Patients with MSI-H in cfDNA (Guardant360^®^, 74 gene next-generation sequencing (NGS) with MBC are identified. We conduct a retrospective review. The median number of alterations and a median maximum mutant allelic fraction (MAF) in MSI-H and non-MSI-H cohorts are compared with Mann–Whitney *U*-test. Of 6718 patients with breast cancer with ≥1 plasma NGS alteration, 42 (0.63%) have MSI-H. A median number of genomic alterations per sample is 11 in MSI-H vs. 3 in non-MSI-H (Mann–Whitney *U*-test *p* < 0.0001) and the median maximum MAF is 16.8% in MSI-H vs. 2.6% in non-MSI-H (Mann–Whitney *U*-test *p* < 0.0001). The co-existing genomic landscape is heterogeneous. The median response duration for seven patients receiving immunotherapy is 92 days (range 29–273 days). CfDNA can identify MSI**-**H in MBC. Research is needed to validate immunotherapy usage in cfDNA-detected MSI-H MBC.

## Introduction

Microsatellite instability (MSI) occurs in many types of tumors from defects in mismatch repair genes, resulting in a mismatch repair deficiency^1–5^. One group identified MSI occurring in 3.8% of cancers assessed, across 39 different cancer types^2^. Tumors with MSI can be further delineated by the amount of instability, as MSI-high (MSI-H) or MSI-low (MSI-L)^6^. Somatic or germline mutations in mismatch repair genes can occur, with both types of alterations associated with MSI-H cancers, and germline mutations can also be found in association with Lynch syndrome^7–9^.

Microsatellite instability can be indicative of hyper-mutated tumors arising from mismatch repair deficiency^4^. Tumors with mismatch repair deficiency have been shown to respond to programmed cell death 1 axis blockade^10,11^, as have tumors with a high mutational burden^12^. Immune checkpoint inhibition is now approved for the treatment of advanced solid tumors with MSI-H and/or mismatch repair deficiency^13^. MSI-H status has typically been determined through the evaluation of tumor tissue^4^.

Plasma-based genotyping is being explored as a complementary methodology to tumor tissue genotyping as it has the advantage of being less invasive than tumor tissue genotyping and can be repeated at the time of disease progression, which may capture a changing genomic landscape under the pressure of treatment^14^. Limitations of this technology include reduced sensitivity in low-shedding tumors^15^. The utility of immunotherapy in MSI-H tumors detected using plasma-based genotyping is currently not known and needs to be understood as plasma-based genotyping assays are being used more commonly in metastatic breast cancer (MBC) to identify targetable mutations^14^.

The objective of this study is to evaluate MSI-H detection using a plasma-based genotyping assay (Guardant360^®^, cell-free DNA (cfDNA), up to 74 gene assay) in a cohort of patients with MBC undergoing plasma-based genotyping, and study the association of MSI-H with clinico-genomic characteristics. We demonstrate that MSI-H status can be identified via cfDNA. MSI-H status correlated with a higher median number of genomic alterations and a medium maximum mutant allelic fraction (MAF). We also retrospectively evaluate the response to immunotherapy in seven patients with MSI-H MBC.

## Results

### Patient characteristics

Of 6718 patients with advanced breast cancer with at least one alteration detected in cfDNA, 42 (0.63%) were MSI-H (with 43 samples).

**Fig. 1:**
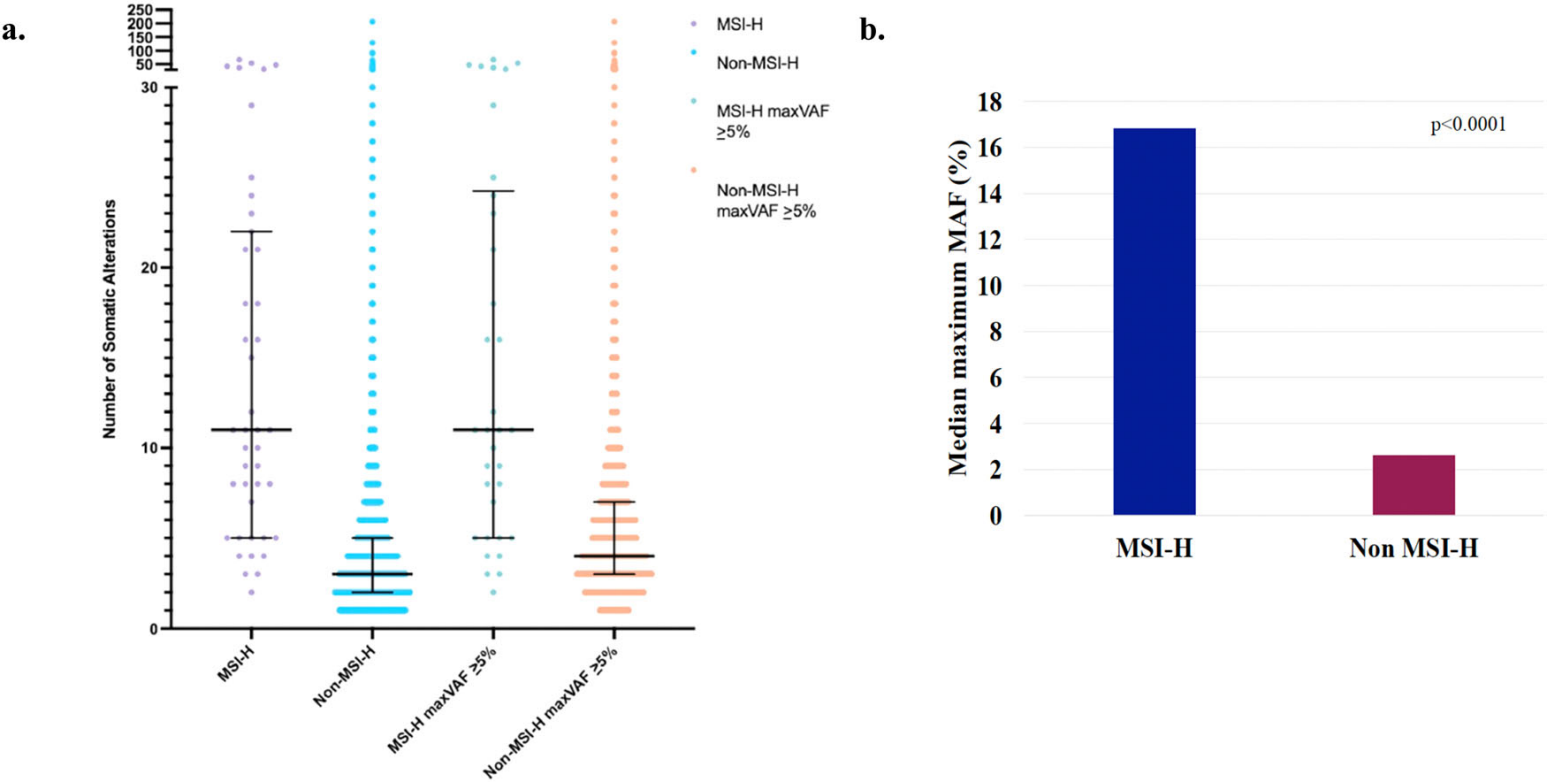
Comparison of the number of somatic alterations and medium maximum MAF in MSI-H versus non-MSI-H samples. **a** Distribution of the number of somatic alterations detected per sample, including synonymous variants and variants of uncertain significance, excluding amplifications, in MSI-H versus non-MSI-H samples. The number of somatic alterations in individual samples is represented by colored dots with median value and interquartile range outlined in black. **b** Median maximum MAF in MSI-H versus non-MSI-H samples (Mann–Whitney *U*-test *p* < 0.0001).

All 42 patients in the MSI-H cohort were female. Their median age was 61 years (range 39–92). As shown in Fig. 1a, the median number of genomic alterations per sample in this MSI-H cohort was 11 (range 2–67) compared to 3 (range 1–206) in the non-MSI-H samples (Mann–Whitney *U*-test *p* < 0.0001). Even when limiting to MSI-H and non-MSI-H samples with a maximum variant allele fraction (maxVAF) of ≥5%, MSI-H samples maintained a significantly higher median number of genomic alterations per sample (11 versus 4 alterations, Mann–Whitney *U*-test *p* < 0.0001). The median maximum mutant allelic fraction (MAF) for the MSI-H cohort was 16.8% (range 0.9–54.2%) compared to 2.6% (range 0.02–96.5%) for the non-MSI-H samples (Mann–Whitney *U*-test *p* < 0.0001) as shown in Fig. 1b.

### Co-existing genomic landscape

**Fig. 2:**
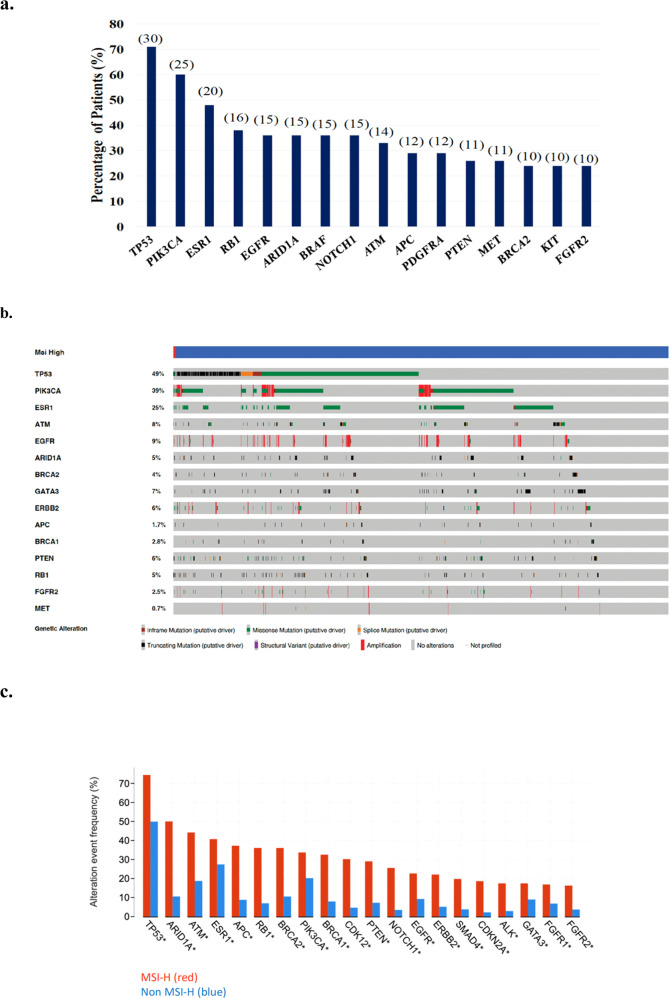
Analysis of co-existing gene mutations. **a** Most common non-synonymous variants seen in MSI-H patients with ≥1 cfDNA alteration detected. Absolute number of patients are indicated in (#). **b** Comparison of 15 most frequent gene mutations (excluding VUS and synonymous variants) in MSI-H samples (red) and non-MSI-H samples (blue). **c** Differences in the frequency of genes (excluding synonymous variants, but including VUS) in MSI-H (red) vs non-MSI-H (blue) samples (* indicating significant differences, *p* < 0.05 one-sided Fisher exact test) of the top 20 significantly mutated genes between groups.

Figure 2A depicts the most common co-existing non-synonymous mutations (including single nucleotide variants (SNVs), indels, and a few amplifications) seen in conjunction with MSI-H in this cohort. As demonstrated in Fig. 2a, the co-existing genomic landscape in patients with MSI-H is heterogeneous, including mutations in *TP53*, *PI3KCA*, *ESR1*, *RB1*, *EGFR*, *ARID1A, BRAF*, *NOTCH1*, DNA damage repair genes (*ATM* and *BRCA2*)*, APC*, *PDGFRA*, *PTEN*, *MET, KIT*, and *FGFR2*. The frequencies of these mutations are indicated in Fig. 2a.

Figure 2b compares the most frequent gene mutations (excluding variants of uncertain significance, VUS, and synonymous variants) in MSI-H and non-MSI-H samples, while statistically significant differences in gene frequencies (excluding synonymous variants) in MSI-H vs. non-MSI-H samples in the top 20 mutated genes between these cohorts are shown in Fig. 2c. Figure 2b, c were generated using cBio Cancer Genomics Portal^16,17^.

### Clinical treatment course

**Fig. 3:**
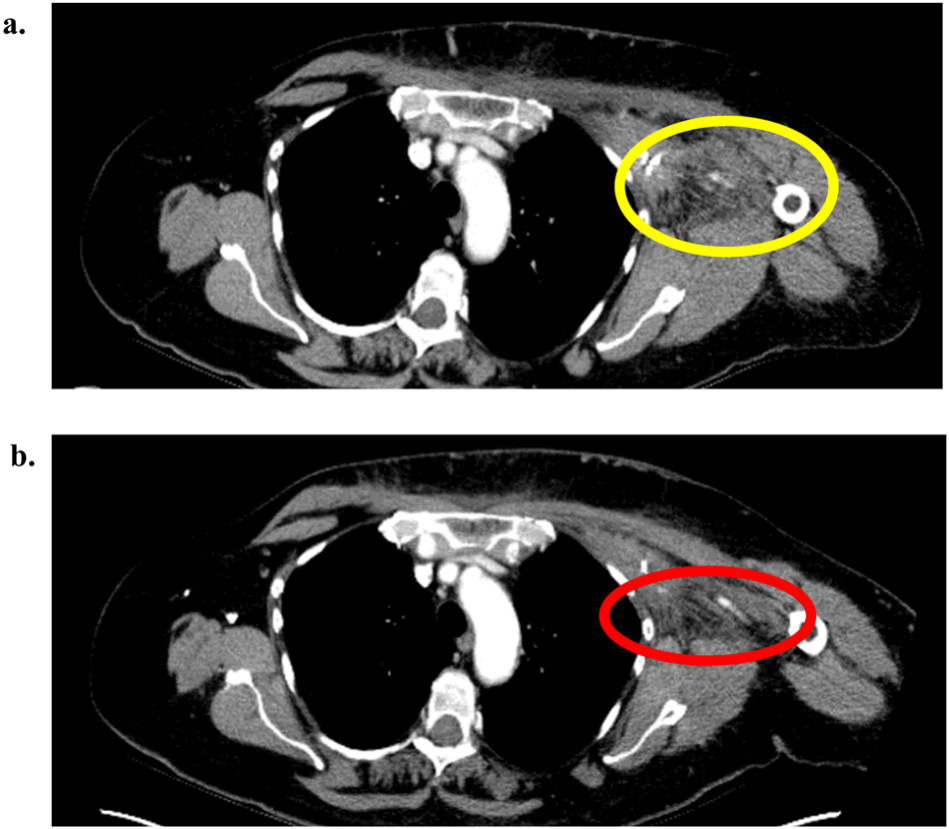
Response to immunotherapy and chemotherapy in a patient with MSI-H positive TNBC (patient ID 1). A decrease in the size of a chest wall metastasis was noted from baseline imaging (**a**) following 5 months of treatment with immunotherapy/chemotherapy (atezolizumab/nab-paclitaxel) (**b**). The patient remained on immunotherapy/chemotherapy for 273 days.

For 11/42 MSI-H patients with MBC, clinical data were available, as summarized in Table 1. Both triple-negative breast cancer (TNBC: 36%) and hormone receptor-positive, HER2 negative (HR+/HER2−) MBC (64%) were seen. None of these 11 patients had a known diagnosis of Lynch syndrome. Two of three patients who had undergone MSI testing of a metastatic tumor tissue specimen also had MSI-H positive disease detected via tissue. Patient 2 had next-generation sequencing (NGS) done of their metastatic liver lesion, which also identified MSI-H. Patient 5 had immunohistochemistry (IHC) testing of the mismatch repair (MMR) genes conducted on a metastatic liver lesion, which identified intact expression of the MMR genes. Patient 6 had tissue NGS testing of a supraclavicular lymph node, which also detected MSI-H. Seven patients underwent treatment with various immune checkpoint inhibitors, as described in Table 2. The median duration of treatment was 92 days (range 29–273 days). There did not appear to be an association between the number of prior lines of therapy and the duration of response, albeit the cohort size was limited. Two patients had a sustained benefit to immunotherapy in combination with chemotherapy, including Patient 1, who was treated with first-line atezolizumab/nab-paclitaxel and experienced stable disease for 273 days (Fig. 3), and Patient 7, who was treated with pembrolizumab/capecitabine followed by pembrolizumab/eribulin in the fifth-line setting who remained on immunotherapy treatment for 245 days; both of these patients did not have prior exposure to either the immunotherapy or chemotherapy agents. Of the two patients whose MSI-H call was concordant with tissue testing, Patient 2 received first-line atezolizumab/nab-paclitaxel and remained on treatment for 91 days, while Patient 6 received fourth-line pembrolizumab monotherapy and remained on treatment for 65 days. Patient 5, who had discordant tissue and plasma MSI-H calls, received tenth-line pembrolizumab monotherapy and remained on treatment for 29 days.

**Table 1:** MSI-H patients with MBC for whom clinical data was available (*N* = 11).

Clinical Characteristic	Analytical result (*N* = 11)
Age at MBC diagnosis	Median: 45
	Range: 34–79
*Subtype*	
Triple-negative breast cancer	4 (36%)
Hormone receptor-positive (HR+)/HER2−	7 (64%)
Known Lynch syndrome	0 (0%)
*Treatment with immune checkpoint inhibitor*	7
Atezolizumab/Nab-paclitaxel	2
Pembrolizumab	3
Pembrolizumab/capecitabine followed by pembrolizumab/eribulin	1
Nivolumab	1
Response to immune checkpoint inhibitor	Median duration of treatment: 92 days (range 29–273 days)

**Table 2:** Characteristics of patients receiving immunotherapy with MSI-H status.

Patient ID	Age at MBC diagnosis	Breast cancer subtype	Co-existing cell-free DNA genomic landscape	Immunotherapy (IO) regimen	IO line of treatment for MBC	Duration of response to IO
1	64	TNBC	*BRCA2, CDKN2A, FGFR1, TP53* VUS: *ALK, APC, ATM, EGFR, MET, MTOR, PIK3CA*	Atezolizumab/nab-paclitaxel	1st	273 days
2	39	TNBC	*KRAS, NOTCH1, PTEN, TP53* VUS: *BRCA2, RIT1*	Atezolizumab/nab-paclitaxel	1st	91 days
3	55	ER+/PR+/HER2−	*APC, ARID1A, BRCA1, CCND1, ESR1, FGFR2, FGFR3, IDH1, KIT, MAP2K1, MTOR, PIK3CA, PTEN, TP53, TSC1* VUS: *AR, ARAF, BRCA2, EGFR, GATA3, HNF1A, KRAS, MAP2K2, MAPK3, MET, NF1, NOTCH1, NRAS, PDGFRA*	Pembrolizumab	6th	108 days
4	37	ER+/PR+/HER2−	*ESR1* VUS: *MAP2K2, NOTCH1*	Nivolumab	5th	92 days
5	34	ER+/PR+/HER2−	*ARID1A, ATM, BRCA1, BRCA2, CCNE1, ESR1, MET, NRAS, PIK3CA, ROS1, TP53* VUS: *APC, AR, BRAF, CDK4, EGFR, GATA3, KIT, MAPK1, MYC, NOTCH1, PDGFRA, SMAD4*	Pembrolizumab	10th	29 days
6	79	ER+/PR−/HER2−	*CCNE1, MTOR, MYC, PIK3CA, PTEN, STK11, TP53* VUS: *ARAF, EGFR, FGFR1, SMAD4, TERT*	Pembrolizumab	4th	65 days
7	45	ER+/PR+/HER2−	*ARID1A, ATM, ESR1, FGFR1, KIT, PIK3CA, TP53*	Pembrolizumab/capecitabine followed by pembrolizumab/eribulin	5th	245 days of immunotherapy (pembrolizumab/capecitabine: 136 days, then pembrolizumab/eribulin: 109 days)

## Discussion

Plasma-based genotyping assays are being used more commonly in MBC, particularly after approval of a targeted therapy, alpelisib, for *PIK3CA* mutant HR+/HER2− MBC^18^. The non-invasive nature of these assays means they can be repeated easily throughout the disease course to capture tumor evolution and may be particularly useful for patients in whom tissue testing is difficult (e.g., for patients with bone-only disease) or not informative (e.g., a “quality not sufficient” result)^14^, although an acknowledged limitation is a potentially reduced sensitivity in low-shedding tumors. Furthermore, tumor tissue biopsies are generally obtained at the time of diagnosis of MBC, and therefore genotyping done on baseline specimens may not necessarily reflect the current genomic environment after treatment with various therapies, which real-time plasma-based genotyping may capture. As plasma-based genotyping is increasingly being used to detect actionable mutations such as *PIK3CA*, the ability to identify other genomic alterations such as MSI-H expands the potential clinical application of these assays.

In this study, we evaluated a plasma-based genotyping assay to identify the presence of MSI-H in advanced breast cancer. In our cohort of 6718 patients with at least 1 detectable mutation found in plasma-based genotyping, MSI-H positive tumors accounted for 0.63%. Our findings suggest that while MSI-H is detectable using a plasma-based assay, it is relatively rare in MBC. Previous studies have suggested an MSI-H frequency in breast cancer ranging from 0–1.5%, aligned with the frequency detected here^2,3,19–21^. In one study of 444 breast cancer samples, IHC of the mismatch repair proteins identified mismatch repair deficiency in 17% of patients. However, when these same 75 samples were tested using a PCR assay utilizing the five indicator sites recommended by the National Cancer Institute, 68/75 (91%) were classified as microsatellite stable^22^. Variations in frequency between all of these studies may reflect differences in how each assay calls MSI-H and differences between the breast cancer cohorts used in each study. Further research is needed to identify the optimal methodology for the detection of MSI-H in breast cancer and validate the clinical utility of plasma-based genotyping in this setting.

In our cohort, MSI-H-positive breast cancers demonstrated a significantly higher number of somatic alterations, even when limited to samples with ≥5% maxVAF. As MSI-H is suggestive of impaired DNA mismatch repair, it seems consistent that MSI-H tumors have significantly more somatic alterations. The prognostic implications of these tumor characteristics in breast cancer need to be further explored. We also observed a wide spectrum of co-existing mutations, including mutations in *TP53, PI3KCA, ESR1, RB1, EGFR, ARID1A*, *BRAF, NOTCH1*, DNA damage repair genes (*ATM* and *BRCA2*), *APC, PDGFRA, PTEN*, *MET, KIT*, and *FGFR2*, consistent with the known genomic complexity of MBC^23^. In addition, MBC may harbor the APOBEC signature in keeping with increasing genomic complexity^23^, and it is not known how MSI-H status correlates with the presence of the APOBEC signature. While there is limited literature on how the APOBEC mutational signature relates to MSI-H, prior studies have observed an increased tumor mutational burden in both MSI-H and APOBEC mutated tumors^24,25^. Understanding the relationship of the APOBEC signature with MSI-H, and how these biomarkers may correlate with response to immunotherapy, is a direction for future research, as we were unable to assess these associations in our cohort.

Although our clinical response cohort is small, it is intriguing to note that some patients had durable responses to treatment with immunotherapy and chemotherapy, including two patients who remained on immunotherapy and chemotherapy for over 245 days. It is also interesting to note that both patients with concordant plasma and tissue MSI-H calls who received immunotherapy (±chemotherapy) remained on treatment for less than 100 days. The patient with discordant plasma and tissue MSI-H calls received pembrolizumab monotherapy for 29 days, although this was the most heavily pretreated patient in the cohort at their tenth-line of therapy. The small sample size, the combination of immunotherapy ± chemotherapy regimens used, and the mixture of lines of therapy make it difficult to interpret the duration of response seen here. However, the fact that a patient who was MSI-H via both plasma and tissue treated with immunotherapy plus chemotherapy in the first-line had a duration of response of only 91 days suggests that further work is needed to better identify patients with MBC who may benefit from immunotherapy.

MSI-H testing has primarily been concentrated amongst the cancers associated with Lynch syndrome^19^. With the expanded use of molecular testing across cancer types, the use of MSI-H as a pan-cancer biomarker is still in its infancy. Notably, the FDA approved the use of pembrolizumab across solid tumors for patients with MSI-H metastatic disease and no satisfactory alternative treatments, meaning that patients with MSI-H MBC could qualify for pembrolizumab regardless of their programmed death ligand 1 (PD-L1) status^13^. However, although the FDA approval applies across solid tumors, many cancer types, including breast, had low or no representation amongst the five KEYNOTE studies used to support this approval^26,27^. For example, the KEYNOTE-158 trial, which contained the largest cohort of non-colorectal MSI-H cancers, included five patients with breast cancer and did not provide a breast cancer-specific response rate^28^. While the utility of immune checkpoint inhibitor therapy in some of these cancer types requires further exploration, preliminary data does support the clinical utility of plasma-based NGS testing in identifying MSI-H patients who may benefit from immune checkpoint inhibitor therapy in this setting. In a study of nine patients with metastatic castration-resistant prostate cancer found to be MSI-H via this same liquid biopsy assay who went on to receive pembrolizumab, four patients (44%) achieved a PSA decrease >50% after a median of 4 weeks, three of whom had a PSA decrease >99%^27^. Among five patients evaluable for response, the overall response rate was 60%, with one complete response and two partial responses^27^.

There is limited data on how MSI status correlates with PD-L1 expression, the current biomarker used to select immunotherapy for MBC, although pan-cancer studies suggest a relatively low correlation between PD-L1 expression and MSI-H status^29^. A prior study evaluating tumor tissue biopsies of TNBC did not identify a correlation between MSI status and PD-L1 expression^30^. Similarly, the overlap between tumor mutational burden (TMB) score and MSI-H has yet to be fully explored in MBC. An analysis done using the same cfDNA assay as in our study compared the frequency of TMB high (defined in a cancer-type-specific manner using the 80th percentile score for mutations/megabase) and MSI-H across multiple cancer types. The frequency of overlap varied significantly, with 7% of breast cancer cases classified as TMB high, also denoted as MSI-H^31^. Recent studies in breast cancer have demonstrated the preliminary efficacy of immunotherapy in tumors with high mutational burden^32,33^. Further exploration of the optimal biomarkers for the selection of patients with MBC who may benefit from immune checkpoint inhibitor therapy is needed. The majority of immunotherapy studies in advanced breast cancer have focused on TNBC as it has a higher level of tumor-infiltrating lymphocytes and PD-L1 expression compared to other breast cancer subtypes. The results of these studies have been mixed, with some patients experiencing a sustained duration of response, but overall low objective response rates^34^. The data available in patients with HR+/HER2− breast cancer is limited, with the KEYNOTE-028 trial of patients with estrogen receptor-positive/HER2- advanced breast cancer and PD-L1 + tumors showing a median progression free survival of 1.8 months and an objective response rate of 12%^35^. Overall, this data suggests that additional research is needed across breast cancer subtypes to better refine treatment selection, including in patients with HR + /HER2- disease, as demonstrated by the patient with HR+/HER2− advanced breast cancer in our study with a duration of response greater than 245 days on pembrolizumab combination therapy.

The study had a few limitations. First, aside from the 11 patients for whom the ordering provider was willing to share additional clinical data, we have limited clinical data for this cohort. As we were reliant on which providers responded to our inquiry, it is possible the cohort may be biased toward providers more interested in MSI-H and, thus, more likely to give immunotherapy in this setting. Of the 11 patients with clinical data available, only three had tissue testing of a metastatic tumor lesion that included orthogonal confirmation of MSI status, a limitation of the retrospective nature of this study. Given the retrospective nature of this study, we were not able to obtain metastatic tumor tissue for MSI-H testing prospectively to evaluate concordance with the blood-based assay. As this study included patients tested from 2018 to 2020, this lack of tissue MSI-H testing may reflect the historic lack of routine testing for MSI-H conducted in patients with breast cancer. Additionally, the version of the assay these patients were tested with included only partial coverage of *MLH1* and did not include *PMS2, MSH2*, or *MSH6*. Thus, we cannot comment on the occurrence of co-occurring pathogenic alterations in these genes in this cohort. The rarity of MSI-H in many cancer types, including breast cancer, means that large cohorts for validation of assay performance by cancer type are difficult; there are technically no validated methods for the detection of MMR status in breast cancer^22^. While the clinical validation cohort used for this assay contained a relatively small number of breast cancer cases, the pan-cancer positive predictive value was 94.7%, and publications examining MSI-H calls made by this assay in other cancer types where MSI-H is rare support the validity of these calls^36,37^. Further exploration of the optimal methodology for the detection of MSI-H in breast cancer is needed. Second, given the limited sample size and retrospective nature of this study, we were not able to assess the clinical utility of immunotherapy in patients with MSI-H MBC, although a few durable responses were seen in this small cohort of seven patients. A prospective study is needed to determine the clinical utility of immunotherapy treatment in MSI-H MBC detected by plasma-based genotyping.

In summary, our work demonstrates that plasma-based genotyping assays can detect MSI-H in breast cancer, including in patients with TNBC and HR+/HER2− MBC. Additional research is needed to further demonstrate the efficacy of immunotherapy in MSI-H MBC and identify the optimal biomarkers for the selection of patients with MBC who may benefit from immunotherapy combinations.

## Methods

### Patient population

A retrospective analysis of a de-identified database containing results from consecutive patients with advanced breast cancer treated within the United States who underwent cell-free circulating tumor DNA (cfDNA) analysis with Guardant360^®^ (next-generation sequencing (NGS), up to 74 gene panels) between 9/27/2018 and 3/12/2020 was conducted. As this database is derived from commercial testing, only internal access from select employees of Guardant Health is permitted. From this subset, the cohort with MSI-H in cfDNA analysis with Guardant360^®^ was identified. Patient age, gender, and cancer type were extracted from the test requisition form. This research was approved by the Quorum Institutional Review Board (IRB) for the generation of de-identified datasets for research purposes. The ordering providers for all 42 MSI-H cases were contacted regarding their interest in participating in this study. Providers responded affirmatively for 11 of these patients, and clinical data were provided by the treating physician to evaluate clinical features and response to immunotherapy. All patients in the dataset had consented (written informed consent) to Guardant360^®^ testing by their treating physician.

### Cell-free DNA analysis

For this study, cell-free circulating tumor DNA (cfDNA) analysis was performed using the Guardant360^®^ assay (Guardant Health, Redwood City, California, USA). Guardant360^®^ is a CLIA-certified, College of American Pathologists-accredited, New York State Department of Health-approved, cfDNA NGS assay, with analytical and clinical validation previously described^38,39^. The assay includes analysis of single nucleotide variants (SNVs) in up to 74 genes (the assay evolved from a 73-gene to a 74-gene panel over the course of the study period), as well as small insertions/deletions (indels), copy number amplifications, and gene rearrangements/fusions in a subset of genes^39^. The genes included in this assay are shown in Supplementary Table 1.

The analytical performance of MSI-H determination via the Guardant360^®^ cfDNA assay has previously been described^40^. Briefly, NGS reads from cfDNA across 90 microsatellite loci are integrated into the MSI-H caller, and samples with a total number of unstable sites exceeding a pre-determined threshold are denoted as MSI-H. Clinical validation of this assay for MSI-H determination using 949 unique patients across 40 different cancer types demonstrated a sensitivity of 86.6% and a specificity of 99.5% in samples with a maximum variant allele fraction in cfDNA ≥0.2%^40^. We note that this clinical validation cohort did rely more heavily on cancers where MSI is more frequent (e.g., colorectal cancer) and included 30 breast cancer samples. However, the positive predictive value of the assay when compared to tissue was 94.7% across cancer types, and publications examining MSI-H calls made by this assay in other cancer types where MSI-H is rare support the validity of these calls^36,37^.

### Statistical analysis

A Mann–Whitney *U*-test was used to compare the median number of alterations per sample and a median maximum mutant allelic fraction (MAF) for the MSI-H cohort and non-MSI-H samples during the study period. A *p* value less than 0.05 was considered statistically significant. All statistical analyses were done using GraphPad Prism version 9.1.0 for macOS, GraphPad Software, San Diego, California, USA, www.graphpad.com.

## Supplementary information

Supplementary Material

Reporting Checklist

## Data Availability

The dataset generated and/or analyzed for the current study are not publicly available as they are derived from commercial testing and can only be shared under a fully executed data use agreement. Inquiries regarding such data use agreements can be made to: medicalaffairs@guardanthealth.com.
