# Supplementary material for: Detection of microsatellite instability high (MSI-H) status by targeted plasma-based genotyping in metastatic breast cancer
Source: NPJ Breast Cancer. 2022 Nov 4;8:117. doi: 10.1038/s41523-022-00490-2 (PMC9636209; doi:10.1038/s41523-022-00490-2)
Supplement: Supplementary file 1 — Supplementary Material [file 41523_2022_490_MOESM1_ESM.pdf]

Vidula N et al.

Supplementary Table 1. Oncogenic mutations included in Guardant360® assay.

**Single nucleotide variants and insertion/deletion alterations**									
AKT1	*ALK*	*APC*	*AR*	*ARAF*	*ARID1A*	*ATM*	*BRAF*	*BRCA1*	*BRCA2*
CCND1	*CCND2*	*CCNE1*	*CDH1*	*CDK4*	*CDK6*	*CDKN2A*	*CTNNB1*	*DDR*	*EGFR*
ERBB2	*ESR1*	*EZH1*	*EZH2*	*FBXW7*	*FGFR1*	*FGFR2*	*FGFR3*	*GATA3*	*GNA11*
GNAQ	*GNAS*	*HNF1A*	*HRAS*	*IDH1*	*IDH2*	*JAK2*	*JAK3*	*KIT*	*KRAS*
MAP2K1	*MAP2K2*	*MAPK1*	*MAPK3*	*MET*	*MLH1*	*MPL*	*MTOR*	*MYC*	*NF1*
NFE2L2	*NOTCH1*	*NPM1*	*NRAS*	*NTRK1*	*NTRK3*	*PDGFRA*	*PIK3CA*	*PTEN*	*PTPN11*
RAF1	*RB1*	*RET*	*RHEB*	*RHOA*	*RIT1*	*ROS1*	*SMAD4*	*SMO*	*STK11*
TERT	*TP53*	*TSC1*	*VHL*						
**Amplifications**									
AR	*BRAF*	*CCND1*	*CCND2*	*CCNE1*	*CDK4*	*CDK6*	*EGFR*	*ERBB2*	*FGFR1*
FGFR2	*KIT*	*KRAS*	*MET*	*MYC*	*PDGFRA*	*PIK3CA*	*RAF1*		
**Fusions**									
ALK	*FGFR2*	*FGFR3*	*RET*	*ROS1*	*NTRK1*				
